# Health and Wellness Tourists’ Motivation and Behavior Intention: The Role of Perceived Value

**DOI:** 10.3390/ijerph20054339

**Published:** 2023-02-28

**Authors:** Ting Gan, Jiansong Zheng, Wei Li, Jiaxin Li, Junxian Shen

**Affiliations:** 1 School of Geography and Tourism, Huanggang Normal University, Huanggang 438000, China; 2 Faculty of Humanities and Social Sciences, Macao Polytechnic University, Macau 999078, China; 3 Education Department, Huanggang Normal University, Huanggang 438000, China; 4 Mental Health Counseling Center, Wuhan Sports University, Wuhan 430079, China

**Keywords:** health and wellness tourism, tourism motivation, perceived value, behavioral intention

## Abstract

In recent years, with the rapid change of people’s health concept, health and wellness tourism has shown a vigorous development trend. However, existing literature has been lacking on travelers’ behavioral intentions, influenced by their motivation in health and wellness tourism. To fill in this gap, we designed scales of tourists’ behavioral intention and motivation in health and wellness tourism and investigated the aforementioned effects, with a sample of 493 visitors who have traveled in health and wellness tourism. Factor analysis and structural equation models were applied to explore the relations among motivation, perceived value, and behavioral intention in health and wellness tourism. The results indicate that health and wellness tourists’ motivation significantly positively predicts their behavior intentions. Travelers’ perceived value of health and wellness tourism significantly partially mediates the associations between their behavioral intention and escape motivation, attractive motivation, environmental motivation, as well as interpersonal motivation. No empirical evidence supports the mediating role of perceived value in the correlation between consumption motivation and behavioral intention. Health and wellness tourism industries are encouraged to meet the intrinsic motivation of travelers and make them perceive the value of this kind of tourism, which in turn promotes tourists’ choice, evaluation, and satisfaction of health and wellness tourism.

## 1. Introduction

Health and wellness tourism has been blooming around the world since the COVID-19 pandemic [1,2,3]. COVID-19 has swept the world and the outbreak has had a major impact on public health and tourism as one of the keys to the global economy [4,5]. The impact of this epidemic on health and wellness tourism is diverse, with the heterogeneity of prevention and control measures in different countries or regions [6]. As a health and economic crisis, the perceived risk of the virus is likely to affect tourists’ intentions to travel [2,7]. The Chinese government has been relaxing a dynamic zero-COVID policy at the end of 2022 [8]. This means that China’s domestic tourism market has been reopened, but the international tourism market is still limited by the epidemic restriction policies of the destination countries or regions. In this context, tourism industries with acceptable prices and positive health orientations may be more popular among travelers [4,7]. Health and wellness tourism has become a powerful catalyst for regional development in a competitive global tourism economy [2,3]. Therefore, it is necessary to explore the motivation and behavioral intentions of tourists toward health and wellness tourism.

As a new kind of integrated development of tourism and health industry, health and wellness tourism’s advantages are outstanding and well-liked by the public, and it has become an emerging form of tourism economy [9,10]. In terms of China’s data, the market size of health and wellness tourism industry reached 82.9 billion yuan in 2019, and the compound annual growth rate of the market size of health tourism has reached about 20% from 2016 to 2020 [11].

The World Tourism Organization considered medical tourism as tourism services with the theme of medical care, rehabilitation, and recuperation [12]. As an advanced concept with medical tourism, health and wellness tourism offers medical and non-medical services to travelers and there are three main definitions of this kind of tourism [13]. The first one considers health and wellness tourism as a comprehensive form of tourism for the purpose of wellness, to obtain physical as well as spiritual relaxation so that people can consciously and enthusiastically participate in various activities in society [13,14]. The second definition considers health and wellness as a way to achieve mental health and physical health by adjusting the body and mind without the need for medical intervention on vacation [15]. Scholars who hold this view believe that tourists are traveling for the purpose of health needs and also to find intangible values, such as a healthy balance of body, mind, and spirit [16]. The third definition is formed through some form of health and wellness tourism and focuses more on the final outcome presented [13,17]. For example, it has been argued that the strength of the visitor’s experience is related to whether the visitor ultimately chooses the destination or not, and this indicator is also effective in predicting the number of visitors in the health and wellness tourism development process [18].

Health and wellness tourism has received extensive attention and research from all walks of life [19]. Most scholars have been concerned with the innovation of health and wellness tourism, the evaluation of health and wellness tourism development resources in case sites, and the corresponding development countermeasures [1,9,13]. However, systematic studies regarding health and wellness tourism are extremely lacking, especially in the field of tourism psychology. Therefore, this study aims to design scales of motivation, perceived value, and behavioral intention in health and wellness tourism, and investigate their relations. The empirical evidence may help industries of health and wellness tourism to design strategies to attract tourists and provide policy implications to tourism sectors of government.

## 2. Literature Review and Hypotheses

### 2.1. Health and Wellness Tourism Motivation and Behavioral Intention

Tourists’ behavioral intentions in health and wellness tourism are able to predict their actual tourism behaviors and can effectively reflect the number of travelers in the tourism market [5,20]. Behavioral intention in health and wellness tourism refers to tourists’ preferences for this kind of tourism [21,22,23]. The high levels of travelers’ behavioral intention represent their preference for health and wellness tourism attractions as destinations, higher satisfaction with the tourism products or services, and willingness to recommend the tour to friends and family [5,24,25]. Tourists’ behavioral intentions are influenced by many factors, including demographic information such as age [26], education level [1,27], health status [28], and psychological factors including motivation [29], risk perception [30], and satisfaction [31]. Existing studies have noted the negative impact of the threat posed by the COVID-19 virus on the tourism market [2,30,32]. However, the COVID-19 epidemic has created a broad market prospect for health and wellness tourism [3,27]. In terms of China, the easing of the containment of the epidemic is a great boon to the domestic tourism industries, especially health and wellness tourism [8,30]. The actual number of the travelers still depends on the behavioral intentions of the individuals, and the motivation to travel is recognized as a determinant of behavioral intentions in health and wellness tourism [29,33].

The push and pull motivation theory can be applied to explain that tourists’ behavioral intentions in health and wellness tourism are driven by motivation [34,35,36]. The push motivation is the intrinsic motivation for people to undertake tourism activities while the pull motivation of tourism is the extrinsic motivation for people to choose a destination for their well-being [36,37,38]. Push motivation can be divided into two sub-dimensions: escape from individuals’ routines [39,40] and acceptable cost [41]. When individuals keep repeating their studies or work, they tend to develop a feeling of dullness and show a sullen state, which is harmful to their health and well-being [19,42]. Escaping from the present environment may heal residents and enable them to relax [43]. Health and wellness tourism with its healing characteristics, is suitable to help working people to relax, when compared to other types of tourism [44,45]. Appropriate spending during tourism can not only be pleasurable, but also satisfy the physical needs of the journey [46,47]. When feeling that the cost of time and money is worthwhile and effective in enhancing their wellness, tourists will show a preference for health and wellness tourism [48]. In this context, travelers may exhibit a greater likelihood of choice for health and wellness tourism. We use the terms escape motivation and cost motivation to describe these psychological processes to explore the influence of push motivation on behavioral intentions in health and wellness tourism [36,49,50]. Based on this, we proposed the hypothesis as follows:

**H1a.**  *Tourists’ escape motivation positively predicts their behavioral intention in health and wellness tourism.*

**H1b.**  *Tourists’ consumption motivation positively predicts their behavioral intention in health and wellness tourism.*

Pull motivations in health and wellness tourism may include the attraction of attractions with wellness features [51], spiritual cleansing from green natural landscapes [52], and the hospitality of friends at the destination or the enhancement of relationships with fellow travelers [53,54]. Three terms including attraction motivation, natural environmental motivation, and interpersonal motivation were used as three sub-dimensions of motivation in health and wellness tourism [53]. Specifically, travelers can be directly healed by purchasing health and wellness tourism products or services including spa, massage, medical product experience, and health care knowledge [13,26,55]. Tourists can enjoy the fresh air in green resorts, which is not available in their routines [52]. Interpersonal interaction has been shown to be a key element in choosing a health and wellness tourism destination [56]. Based on this, we proposed the hypothesis as follows.

**H1c.**  *Tourists’ attractive motivation positively predicts their behavioral intention in health and wellness tourism.*

**H1d.**  *Tourists’ natural environmental motivation positively predicts their behavioral intention in health and wellness tourism.*

**H1e.**  *Tourists’ interpersonal motivation positively predicts their behavioral intention in health and wellness tourism.*

### 2.2. Perceived Value as a Mediator

Tourists’ perceived value refers to the result of a comparison between travelers’ costs in their travel process and what they acquire after an actual tour [57,58]. Tourists’ perceived value is a determining factor in their choice, evaluation, and satisfaction with regard to particular attractions [24,32,58,59]. When travelers perceive the value of health and wellness tourism, they will give extra consideration to the health-enhancing effects brought by this type of tourism [54,60]. In general, perceived value can be subdivided into some dimensions including functional, emotional, and social perceptions, as well as perceived sacrifice [24,61,62,63]. In terms of health and wellness tourism, tourists’ perceived value can be considered as health perceived value, emotion perceived value, and cost perceived value [13,64]. When travelers perceive high quality of health and wellness products or services, they may show higher behavioral intentions in this kind of tourism [24,65].

Tourists with the push motivation may perceive the high value of health and wellness tourism [13,42]. For instance, individuals desire to escape their routines when perceiving the health and emotional value of health and wellness tourism [66]. When it is felt that health and wellness tourism cost including time and money is acceptable, travelers may be able to generate a sense of worthiness towards the tourism [46,47]. The pull motivation of travelers also may positively influence their perceived value of health and wellness tourism [13,37]. Individuals may be attracted by health and wellness products or services and a healthy environmental atmosphere, and for the sake of their health status improvement, they likely perceive a high value of health and wellness tourism [42,54,66]. Interpersonal motivation is an antecedent to holistic tourism and may also play a role in the choice of health and wellness tourism, especially when there are older adults or chronic disease patients in the tour groups [42,66,67]. Based on this, we propose the hypothesis as follows.

**H2a.**  *Tourists’ escape motivation has a positive impact on their perceived value of health and wellness tourism, which, in tune, positively predicts their behavioral intentions.*

**H2b.**  *Tourists’ consumption motivation has a positive impact on their perceived value of health and wellness tourism, which, in tune, positively predicts their behavioral intentions.*

**H2c.**  *Tourists’ attraction motivation has a positive impact on their perceived value of health and wellness tourism, which, in tune, positively predicts their behavioral intentions.*

**H2d.**  *Tourists’ natural environmental motivation has a positive impact on their perceived value of health and wellness tourism, which, in tune, positively predicts their behavioral intentions.*

**H2e.**  *Tourists’ interpersonal motivation has a positive impact on their perceived value of health and wellness tourism, which, in tune, positively predicts their behavioral intentions.*

**Figure 1:**
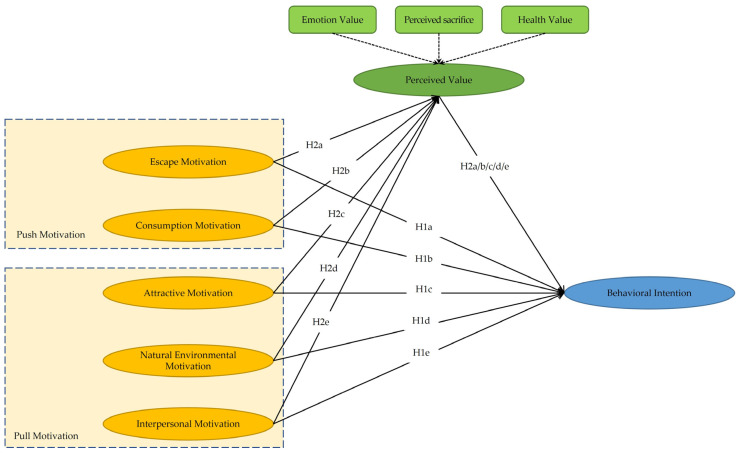
The Framework.

Based on the theory of push and pull motivation, we explored tourists’ behavioral intention as regards tourism in line with tourism motivation, including escape, consumption, attractive natural environmental, and interpersonal motivation in health and wellness tourism. We focused on the mechanism including the mediating effect of travelers’ perceived value on the aforementioned relations. We designed scales for the variables including behavioral intention, tourism motivation, and perceived value, and methods including Cronbach’s *α* value, Kaiser–Meyer–Olkin value, Bartlett’s sphericity test, and exploratory factor analysis were used to ensure the reliability and validity of these scales. Confirmatory factor analysis and the structural equation model were used to explore the effects of tourists’ push and pull motivation on their behavioral intentions in health and wellness tourism and the intermediary mechanisms, with perceived value as the mediating variable. The study framework is shown in Figure 1.

## 3. Methodology

### 3.1. Data Source

The survey was conducted from June to September 2022 and the respondents were people who have participated in health and wellness tourism in China at least once. With the help of travel agency staff, online questionnaires were distributed to the tourists. We briefed the participants on the purpose of the questionnaire and they were asked to answer voluntarily. A total of 510 questionnaires were distributed, and 493 valid questionnaires were collected, with an effective rate of 96.7%. The samples were obtained from many provinces in China, including Sichuan, Guizhou, Hubei, Hunan, Shandong, Guangdong, Tianjin, Beijing, Jiangsu, Zhejiang, Guanxi, Liaoning, Jilin, Heilongjiang, Anhui, Henan, Shanxi.

### 3.2. Variables

**Behavioral intention** Referring to Sthapit, Del Chiappa, Coudounaris, and Björk [21], six items were utilized to portray tourists’ behavioral intentions in health and wellness tourism, as shown in Table 1. Respondents were asked to answer how they approved of these items using a 5-point Likert scale and a higher score represents travelers’ higher behavioral intention in health and wellness tourism. In this study, the Cronbach’s α coefficient of behavioral intention was 0.897.

**Escape motivation** Referring to Wong, Musa, and Taha [50] and Lee and Li [53], three items were designed to reflect the escape motivation of tourists as shown in Table 1. The participants filled in the degree of agreement with the items using a 5-point Likert scale, with a higher score representing a higher level of escape motivation. In this study, the Cronbach’s *α* coefficient of escape motivation was 0.800.

**Attractive motivation** Referring to Wong, Musa, and Taha [50] and Lee and Li [53], three items were used to indicate the attractive motivation of tourists, as shown in Table 1. The participants filled in the degree of agreement with the items using a 5-point Likert scale, with a higher score representing a higher level of attractive motivation. In this study, the Cronbach’s *α* coefficient of attractive motivation was 0.812.

**Consumption motivation** Referring to Wong, Musa, and Taha [50], three items were used to indicate consumption motivation of tourists, as shown in Table 1. The participants filled in the degree of agreement with the items using a 5-point Likert scale, with a higher score representing a higher level of consumption motivation. In this study, the Cronbach’s *α* coefficient of consumption motivation was 0.822.

**Natural environmental motivation** Referring to Wong, Musa, and Taha [50] and Lee and Li [53], three items were used to represent natural environmental motivation of tourists, as shown in Table 1. The participants filled in the degree of agreement with the items using a 5-point Likert scale, with a higher score representing a higher level of consumption motivation. In this study, the Cronbach’s *α* coefficient of consumption motivation was 0.812.

**Interpersonal motivation** Referring to Wong, Musa, and Taha [50], five items were used to indicate interpersonal motivation of tourists, as shown in Table 1. The participants filled in the degree of agreement with the items by using a 5-point Likert scale, with a higher score representing a higher level of consumption motivation. In this study, the Cronbach’s *α* coefficient of consumption motivation was 0.864.

**Perceived value** Referring to Dai, Zhao, Wang, and Zeng [58] and Lee and Li [53], tourists’ perceived value of health and wellness tourism was divided into three sub-dimensions, including emotional value, perceived sacrifice, and health value, and the corresponding items were designed, as shown in Table 1. Emotional value was measured by four items using a 5-point Likert scale. A higher score represents higher levels of perceived emotional value of tourists. Perceived sacrifice was assessed by three items using a 5-point Likert scale. A higher score means more acceptable monetary aspects, such as price, and non-monetary aspects, such as time, convenience, and physical efforts, that tourists perceived. Health value was estimated by five items using a 5-point Likert scale. A higher score represents higher degrees of perceived health value of tourists. In this study, the Cronbach’s *α* coefficients of perceived emotion, cost, health, and the total value were 0.873, 0.814, 0.859, and 0.910, respectively.

## 4. Results

### 4.1. Descriptive Statistics

Table 2 shows the demographic information of the respondents. Among them, over half (58.4%) were female. A larger proportion (70%) of the respondents were aged between 18 and 45, meaning that health and wellness tourism tourists are getting younger. Nearly 90% of the participants had a junior college education or above. Over half (59.3%) of the respondents earned 3000 to 10,000 yuan per month. Over half (55.0%) of the participants were company employees. All individuals have traveled for health and wellness tourism at least once.

### 4.2. Analysis of Validity and Reliability

#### 4.2.1. Exploratory Factor Analysis

The Cronbach’s *α* coefficients of behavioral intention, five sub-dimensions of tourism motivation, and perceived value were all above or equal to 0.800, meaning that the validity of these variables was good [68]. By using SPSS 22.0, the Kaiser–Meyer–Olkin (KMO) value was calculated and Bartlett’s test was applied. The results showed that the KMO values of behavioral intention, tourism motivation, and perceived value were 0.905, 0.869, and 0.919, respectively, while the approximate square card of behavioral intention, tourism motivation, and perceived value were 3057.033 (*df* = 66, *p* < 0.001), 3782.221 (*df* = 136, *p* < 0.001), and 1581.617(*df* = 15, *p* < 0.001), respectively. The KMO values were all around 0.900, while Bartlett’s tests were accepted [69]. The reliability was good. Exploratory factor analysis can be utilized for the collected data. Principle factor analysis was used to perform exploratory factor analysis. If characteristic root values of principal components were greater than 1, the principal components were extracted [70]. The maximum variance method was used to rotate the factors [70].

Table 1 and Table 3 showed the results of the exploratory factor analysis (If the factors were not extracted, their factor loadings were not listed. However, the details are available to readers upon request from the authors). The results showed that only one component could be extracted from tourists’ behavioral intentions in health and wellness tourism. The eigenvalue of this factor was 3.964, and the variance explanation rates reached 66.071%. In terms of tourists’ motivation scale, five factors were extracted, and these factors were escape, consumption, attractive, natural environmental, and interpersonal motivation, and the variance explanation rates of these five factors after rotation were 18.985%, 13.255%, 13.009%, 12.945%, and 12.664%, respectively, and the cumulative variance explanation rate after rotation reached 70.857%. Three factors were extracted from the perceived value scale. The three factors of perceived value were emotion, cost, and health value, and the explained variances of these three factors after rotation were 25.656%, 24.262%, and 19.523%, respectively, and the cumulative explained variance after rotation was 69.441%. The results showed that the factor loadings were all greater than 0.5, meaning that they all met the internal consistency reliability requirements [70,71].

We calculated the statistical characteristics of the items, and these characteristics included mean, standard deviation, skewness, and kurtosis, as shown in Table 1. The mean scores of each variable are between 3 and 5 on 5-point Likert scales, indicating that the level of participants’ tourism motivation, perceived value, and behavioral intention in health and wellness tourism were all above the medium level. Skewness and kurtosis of measurement items were used to ensure the normality of the data. According to the criteria proposed by Kline [72], if the absolute value of the skewness value is within 3 and the kurtosis value is within 8, the data can be considered to satisfy the requirements of an approximately normal distribution [73]. The absolute values of skewness and kurtosis coefficients of each measurement question item in this study were within the standard range (Table 1). Therefore, the data conformed to an approximate normal distribution.

#### 4.2.2. Confirmatory Factor Analysis

(1) Aggregation validity of the measurement model

Confirmatory factor analysis was utilized to verify the validity of the structural equation model. The model fit was good according to the ideal fit criteria (Table 3) [74]. The standardized loading coefficients for each observed item were significantly greater than 0.5, implying a high level of correlation between each dimension and its constituent latent variables (Table 1) [70]. The construct reliabilities (CR) of the latent variables were all greater than 0.7 and their average variance extractions (AVE) were all greater than 0.5, indicating high aggregation validity between the variables (Table 1) [71]. The variance inflation factors (VIFs) of the question items were all less than 3, and there was no significant multicollinearity among the items [70].

(2) Discriminant validity of the measurement model

The correlation coefficients between the study variables were compared with the square roots of their AVEs. The discriminant validity was good when the square root of AVE of a variable is smaller than its correlation coefficient with other variables [75]. There was a higher discriminant validity in this study, and a path analysis of the structural equation model can be applied (Table 4).

### 4.3. Results of Structural Equation Model

**Figure 2:**
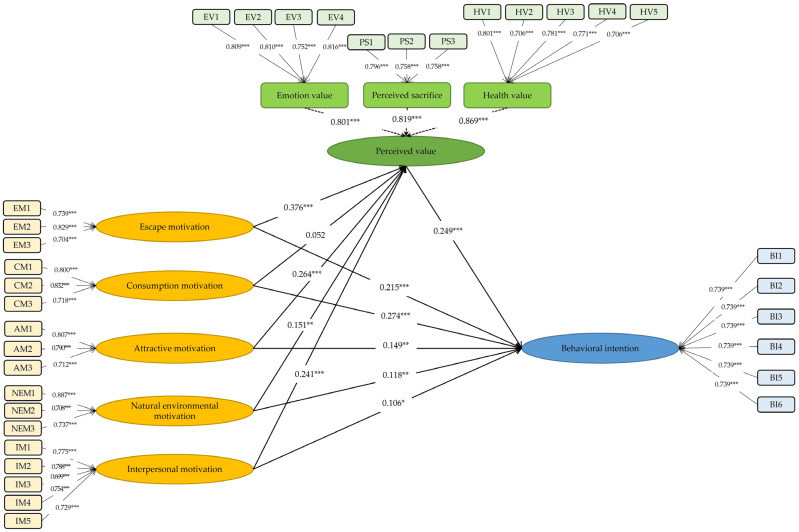
Result of structural equation model. Notes. N = 493; * *p* < 0.05, ** *p* < 0.01, *** *p* < 0.001.

Amos 26.0 was applied to solve the structural equation model. Figure 2 shows the results of the structural equation model. In the structural equation model verification, CMIN was 814.554, CMIN/*df* was 1.520, GFI, AGFI, NFI, RFI, IFI, TLI, and CFI were all above 0.9, RMSEA was 0.033, less than 0.08, Standardized RMR was 0.035, less than 0.05 [76]. Almost all the fitting indexes were in line with the standards of a general structural equation model, and therefore, the model fits well [74].

Table 5 depicted the influencing paths of the variables. Tourists’ behavioral intentions were significantly positively predicted by tourism motivation, including escape motivation, consumption motivation, attractive motivation, natural environmental motivation, and interpersonal motivation (*β* = 0.215, *p* < 0.001; *β* = 0.274, *p* < 0.001; *β* = 0.149, *p* = 0.002; *β* = 0.118, *p* = 0.004; *β* = 0.106, *p* = 0.030, respectively). Tourists’ perceived value was significantly positively predicted by escape motivation, attractive motivation, natural environmental motivation, and interpersonal motivation (*β* = 0.376, *p* < 0.001; *β* = 0.264, *p* < 0.001; *β* = 0.151, *p* = 0.001; *β* = 0.241, *p* < 0.001, respectively). Travelers’ consumption motivation did not have a significant impact on their perceived value in health and wellness tourism (*β* = 0.052, *p* = 0.273). Travelers’ perceived value significantly positively predicted their behavioral intention in health and wellness tourism (*β* = 0.249, *p* = 0.002).

The bias-corrected bootstrap method with 5000 samples was utilized to verify the mediating effect of perceived value on the relations between behavioral intention and tourism motivation, including escape, consumption, attractive, natural environmental, and interpersonal motivation. The results showed that tourists’ perceived value significantly partially mediates the association between behavioral intention and escape, attractive, natural environmental, and interpersonal motivation, and the indirect effects accounted for 30.2%, 30.4%, 24.4%, and 36.4% of the total effect (*p*s < 0.05 and the 95% confidence interval includes 0, Table 6). H2a, H2c, H2d, and H2e were tested. The indirect effect of “consumption motivation → perceived value → behavioral intention” was insignificant (*p* = 0.199 and the 95% confidence interval does not include 0, Table 6), indicating that tourists’ perceived value was not a significant mediator in the relation between consumption motivation and behavioral intention. H2b was not supported.

## 5. Conclusions, Discussion, and Implications

### 5.1. Conclusions

Recently, health and wellness tourism has been developing, especially in China, possibly due to the relaxation of COVID-19 prevention and control restrictions [2,8,13]. However, few studies have focused on tourists’ psychological variables, including motivation, perceived value, and behavioral intention in health and wellness tourism. Based on the theory of push and pull motivation, factor analysis and structural equation models were utilized to explore the relations between tourism motivation and behavioral intention in health and wellness tourism, and the mediating role of perceived value in the aforementioned relation. The results showed that (i) tourists’ motivation significantly positively predicted their behavioral intention in health and wellness tourism and (ii) perceived value significantly played a partial mediating role in the association between tourists’ motivation and behavioral intention in health and wellness tourism. In particular, the four sub-dimensions of tourists’ motivation, including escape motivation, attractive motivation, natural environmental motivation, and interpersonal motivation significantly positively impacted their perceived value, which in tune significantly positively impacted their behavioral intention, while no empirical evidence supported the mediation effect of perceived value on the correlation between behavioral intention and consumption motivation.

### 5.2. Discussion

Empirical evidence indicated that tourists’ push motivation, including escape and consumption motivation, have greater levels of direct influence on their behavioral intention in health and wellness tourism. The important reason for travelers to start a journey is that people are currently facing stress in their work, family, and other aspects, and they want to forget the worries of real life by escaping from the original environment, and health and wellness tourism can precisely meet their needs in this regard [49,50]. In future development, health and wellness tourism scenic spots should strive to create a comfortable, relaxing, and pleasant healing atmosphere and let travelers feel a healthy environment different from their routines [13]. The empirical findings showed that the acceptable sacrifice is also one of the key reasons for travelers to consider choosing health and wellness tourism. Health and wellness tourism enterprises can pay special attention to the cost expenditure of tourists, and moderate concessions of benefits to them, so that they really feel value for money in tourism [58].

The finding supported the significant positive impacts of travelers’ pull motivation on their behavioral intentions in health and wellness tourism, although these direct effects were relatively weaker than the influences of push motivation. This is similar to conclusions of the related literature [29,34,77]. Health and wellness tourism industries should focus more on the quality of products or services to meet the intrinsic needs of travelers, such as escaping their daily routine [78,79]. Health and wellness tourism companies are encouraged to invest more in the construction of tourism products or services rather than in promotion and publicity [9,13,80].

Tourists’ perceived value plays a significant mediating role in the relations between behavioral intention in health and wellness tourism and escape, attractive, natural environmental, interpersonal motivation. When travelers perceive the value of health and wellness tourism, including emotion value, perceived sacrifice, and health value, they will be more likely to choose health and wellness tourism, driven by their tourism motivation [58,62,63]. Health and wellness tourism industries can promote the benefits of products or services, such as health maintenance, wellness, and rehabilitation, to attract people and capture the market [13,63,81]. No empirical evidence supported the mediating role of perceived value in the association between tourists’ behavioral intention and consumption motivation as the possible reason that the perceived sacrifice of visitors does not necessarily correlate with their perceived value of health and wellness tourism [30,42]. Tourists, especially the wealthy group, may consider that health is priceless [82]. In this case, health and wellness tourism companies can set up mass and high-end products or services targeting different income groups.

Higher levels of tourist participation cannot only attract tourists to the tourist destination, but can also be closely related to the tourists’ perceived value and satisfaction evaluation [81]. The positive experiential feel of tourism allows travelers to experience the local culture, and in this case, they can tap the freshness and fun of the trips [55,83]. Health and wellness tourism is easily embedded in an experiential style of tourism [18,84]. Therefore, scenic spots can increase the deep integration of sports, health activities, and study activities beneficial to physical and mental health for different groups [13]. For example, for the parent–child market, forest adventure activities and family camping camps can be launched [85,86]; for student groups, field mini-lecture hall activities can be held to expand outdoor knowledge [85,87]; for older groups, health herbal classes and activities such as tea tasting and health meals sharing sessions can be organized [50]. These activities can enhance the participation of different visitor groups and enrich their health and wellness tourism experience, which, in turn, may improve their revisitation rate and willingness to recommend health and wellness tourism.

### 5.3. Implications

This study investigates tourists’ behavioral intention in health and wellness tourism from the perspective of their push and pull motivation, which may fill the gap in existing theories. In-depth research on tourists’ mindsets and choices can improve the theoretical utility of health and wellness tourism. Tourists participating in health and wellness tourism often pursue the experience of restoring body and mind, which reflects their special psychological needs and behavioral characteristics [18,21]. Understanding and grasping tourists’ motivation and perceived value is actually focusing on tourists’ psychological condition, so as to develop targeted health and wellness tourism products or services [59]. In this vein, personalized tourism services can be provided and the healthy development of health and wellness tourism can be truly realized [13,14]. In addition, paying attention to tourists’ tourism psychological activities can enrich the theoretical system of sociology and psychology in the study of tourism experience value theory. All in all, the empirical findings can provide references for future relevant policies and construction at the government level and tourism enterprise level, and, to a certain extent, complement the theoretical study of health and wellness tourism.

## 6. Limitations

There are several limitations to this study. Firstly, this paper mainly employed factor analysis and structural equation modeling to study the influencing mechanism, but we did not test the causality between tourists’ behavioral intention and tourism motivation, although we explored the mediating role of perceived value in the aforementioned effect. Secondly, over half of the respondents were company employees, implying a lower heterogeneous characterization of the sample with occupation classification. However, company employees have travel spending power, are prone to travel for relaxation, and have a greater propensity for health and wellness tourism. They can be the main target audience for the design of health and wellness tourism products.

## Figures and Tables

**Table 1:** Measurement items and statistical characteristics of the variables.

Variables	Dimensions	Codes	Measurement Items	Mean	Std. Dev.	Skewness	Kurtosis	Factor Loading	AVE	CR	VIF
Behavioral Intention	-	BI1	I will continue to participate in health and wellness tourism.	3.860	0.999	−0.840	0.436	0.802	0.661	0.900	2.075
		BI2	Health and wellness will be my first choice for future trips.	3.820	1.125	−1.013	0.411	0.858			2.623
		BI3	I would choose health and wellness tourism even if the cost is going up.	3.860	1.084	−1.029	0.550	0.806			2.103
		BI4	I will actively promote health and wellness tourism to my family, friends, and colleagues.	3.840	1.127	−0.955	0.264	0.847			2.500
		BI5	I will actively recommend health and wellness tourism to people in the neighborhood.	3.890	1.167	−1.087	0.502	0.797			2.023
		BI6	When someone comes to me for travel advice, I recommend health and wellness tourism.	3.800	1.085	−0.988	0.505	0.763			1.809
Tourism Motivation	Escape motivation	EM1	I am there to feel the slow pace of life.	4.130	0.904	−1.394	2.270	0.727	0.804	0.578	1.617
		EM2	I am there to relieve stress.	4.150	0.955	−1.386	2.020	0.814			1.966
		EM3	I am trying to escape the worries of real life for a while.	4.240	0.949	−1.274	1.299	0.807			1.691
	Consumption motivation	CM1	It is easy to get around here.	4.060	1.060	−1.153	0.680	0.812	0.828	0.616	1.958
		CM2	The cost of transportation is within acceptable limits.	3.970	0.978	−0.842	0.008	0.877			2.223
		CM3	The local consumption level is appropriate.	4.030	0.969	−0.933	0.382	0.756			1.646
	Attractive motivation	AM1	I get word-of-mouth recommendations from friends and family.	4.160	1.039	−1.234	0.713	0.843	0.814	0.594	1.982
		AM2	I am attracted by the promotion of online travel platforms, advertisements, etc.	4.050	0.938	−1.225	1.438	0.773			1.811
		AM3	I am attracted by the sharing of other people’s tour experiences on social media platforms such as WeChat, Weibo, and short videos.	4.090	0.958	−1.176	0.930	0.808			1.656
	Natural environmental motivation	NEM1	The local climate is good and the temperature is comfortable.	4.000	1.069	−1.155	0.916	0.873	0.827	0.612	2.355
		NEM2	The air is fresh here.	4.060	1.060	−1.062	0.485	0.785			1.701
		NEM3	This place can be relaxing for the mind and body.	4.070	0.951	−0.871	0.118	0.784			1.792
	Interpersonal motivation	IM1	I am here to spend time with my family.	3.890	1.158	−0.871	0.019	0.752	0.824	0.562	1.996
		IM2	I am here to visit friends and family in the neighborhood.	4.080	1.061	−1.091	0.576	0.730			2.044
		IM3	I come here to improve my relationship with my companions.	3.930	0.981	−0.795	0.234	0.756			1.748
		IM4	I am here to share my travel experiences with others and gain social acceptance after my trip.	3.980	1.070	−1.137	0.935	0.777			1.998
		IM5	I am here to make new friends and expand my social circle.	4.090	0.972	−1.344	1.698	0.773			1.842
Perceived Value	Emotional value	EV1	I felt the good recreational environment here.	3.880	1.178	−1.050	0.218	0.777	0.874	0.636	2.379
		EV2	I obtained a relaxed mood.	3.660	1.279	−0.852	−0.326	0.818			2.364
		EV3	I temporarily forgot the troubles of real life.	3.890	1.224	−0.995	−0.012	0.758			2.002
		EV4	I experienced pleasurable feelings.	3.780	1.189	−0.936	−0.146	0.800			2.442
	Perceived sacrifice	PS1	The travel time for this trip was acceptable.	3.750	1.218	−0.946	−0.067	0.799	0.815	0.595	2.085
		PS2	The overall consumption level of this place was acceptable.	3.850	1.247	−1.015	−0.022	0.820			1.902
		PS3	The accommodation, food, and shopping were convenient and cost-effective.	3.960	1.163	−0.910	−0.311	0.746			1.891
	Health value	HV1	My body got a workout.	3.970	1.049	−1.070	0.759	0.741	0.859	0.551	2.318
		HV2	My physique was improved.	3.880	1.057	−0.996	0.593	0.751			1.901
		HV3	I recognized the importance of a healthy lifestyle.	3.900	1.001	−1.072	1.108	0.698			2.226
		HV4	I recognized the importance of good eating habits.	3.970	1.017	−0.926	0.309	0.733			1.885
		HV5	My overall personal state has been adjusted.	3.910	1.058	−0.988	0.530	0.697			1.882

Notes. “-” represents no dimension of behavioral intention.

**Table 2:** Demographic Profile of Respondents.

Indices	Items	Numbers	Percentage (%)
Gender	Female	205	41.6
	Male	288	58.4
Age	18–30	173	35.1
	31–45	172	34.9
	46–60	119	24.1
	>60	29	5.9
Education level	High school and below	45	9.1
	Junior college education	110	22.3
	Undergraduate	274	55.6
	Postgraduate	64	13.0
Monthly income (RMB)	<3000	72	14.6
	3001–5000	90	18.3
	5001–10,000	202	41.0
	>10,000	129	26.2
Occupation	Student	45	9.1
	Technician/academician	43	8.7
	Medical Staff	16	3.2
	Farmer	16	3.2
	Freelancer	28	5.7
	Worker	33	6.7
	Business/Company Staff	271	55.0
	Official from government	18	3.7
	Individual operators	21	4.3
	Other	2	0.4

Notes: N = 493.

**Table 3:** Model fitness test results.

Indicators	Reference Standards	Results
CMIN/*df*	1–3 is excellent,	1.473
	3–5 is good	
RMSEA	<0.05 is excellent, <0.08 is good	0.031
IFI	>0.9 is excellent,	0.972
	>0.8 is good	
TLI	>0.9 is excellent,	0.969
	>0.8 is good	
CFI	>0.9 is excellent,	0.972
	>0.8 is good	

**Table 4:** Discriminant validity of measurement models.

Variables	1	2	3	4	5	6	7
1. Behavioral intention	(0.813)						
2. Escape motivation	0.598 ***	(0.897)					
3. Consumption motivation	0.572 ***	0.368 ***	(0.910)				
4. Attractive motivation	0.546 ***	0.407 ***	0.388 ***	(0.902)			
5. Natural environmental motivation	0.478 ***	0.391 ***	0.261 ***	0.295 ***	(0.909)		
6. Interpersonal motivation	0.570 ***	0.469 ***	0.409 ***	0.386 ***	0.420 ***	(0.908)	
7. Perceived value	0.665 ***	0.597 ***	0.420 ***	0.523 ***	0.468 ***	0.558 ***	(0.710)

Note. ***, *p* < 0.001; The value in parentheses refers to the square root of AVE of the corresponding variable.

**Table 5:** Pathways analysis.

Influencing Pathways	Standardized Coefficients	S.E.	*p*
Escape motivation → Behavioral intention	0.215	0.064	<0.001
Consumption motivation → Behavioral intention	0.274	0.046	<0.001
Attractive motivation → Behavioral intention	0.149	0.052	0.002
Natural environmental motivation → Behavioral intention	0.118	0.043	0.004
Interpersonal motivation → Behavioral intention	0.106	0.051	0.030
Escape motivation → Perceived value	0.376	0.069	<0.001
Consumption motivation → Perceived value	0.052	0.052	0.273
Attractive motivation → Perceived value	0.264	0.059	<0.001
Natural environmental motivation → Perceived value	0.151	0.051	0.001
Interpersonal motivation → Perceived value	0.241	0.060	<0.001
Perceived value → Behavioral intention	0.249	0.076	0.002

**Table 6:** Mediation analysis.

Mediation Paths	Category of Effect	Standard Estimate	S.E.	95% Confidence Interval	
				LLCI	ULCI
EM→PV→BI	Direct effect	0.238	0.060	0.132	0.371
	Indirect effect	0.103	0.033	0.055	0.186
	Total effect	0.341	0.055	0.247	0.465
CM→PV→BI	Direct effect	0.291	0.056	0.191	0.417
	Indirect effect	0.014	0.014	−0.008	0.047
	Total effect	0.305	0.057	0.204	0.427
AM→PV→BI	Direct effect	0.162	0.043	0.790	0.249
	Indirect effect	0.071	0.027	0.033	0.146
	Total effect	0.233	0.043	0.151	0.323
NEM→PV→BI	Direct effect	0.125	0.034	0.054	0.187
	Indirect effect	0.040	0.020	0.009	0.090
	Total effect	0.164	0.036	0.087	0.230
IM→PV→BI	Direct effect	0.111	0.042	0.026	0.190
	Indirect effect	0.063	0.029	0.023	0.146
	Total effect	0.173	0.041	0.093	0.253

Notes. EM represents escape motivation; CM represents consumption motivation; AM represents attractive motivation; NEM represents natural environmental motivation; IM represents interpersonal motivation; PV represents perceived value; BI represents behavioral intention. LLCI and ULCI mean lower and upper confidence interval limits, respectively.

## Data Availability

Data connected to this research are available from the corresponding author under request.
